# Comparison of Stigmatization of Suicidal People by Medical Professionals with Stigmatization by the General Population

**DOI:** 10.3390/healthcare9070896

**Published:** 2021-07-15

**Authors:** Jill Julia Eilers, Erich Kasten, Thomas Schnell

**Affiliations:** Department of Psychology, Faculty of Human Sciences, Medical School Hamburg, 20457 Hamburg, Germany; erikasten@aol.com (E.K.); thomas.schnell@medicalschool-hamburg.de (T.S.)

**Keywords:** suicide, suicidality, stigmatization, healthcare professionals

## Abstract

Stigmatization of suicide (SOS) affects help-seeking for suicidality and impedes successful treatment. This study aimed to identify different types of stigmatization and understand the causes and glorification of suicide by comparing three groups; within each of the following groups, the impact of age and gender was explored: (1) practicing medical professional in direct contact with suicidality (psychotherapists, psychiatrists, related medical professions (nurses, etc.)), (2) future medical professionals still in training, (3) and the general population with no professional contact with suicidality. German adults completed an online survey with a total of 742 participants. A MANCOVA was calculated with age and gender being controlled as covariates, due to different distribution. Practicing professionals showed significantly higher levels of SOS than the other groups, while the future professionals showed no differences in SOS from the general population. The understanding of suicide causes was similar across all groups. Men showed higher levels of SOS than women, while women scored higher at understanding of causes and glorification of suicide. Within the individual groups, female professionals in the age group “36–65 years” stigmatized suicide most, while showing the least glorification. The results suggest that tendencies towards SOS are promoted by practical experience with suicidality. Therefore, special training is recommended to reduce SOS.

## 1. Introduction

Suicide is a significant public health issue associated with a high level of suffering. About 800,000 people worldwide commit suicide every year—10,000 of them in Germany. This exceeds the total number of deaths caused by traffic accidents, drug abuse, and HIV in Germany [1].

Over the past 45 years, the number of suicides increased by 60%, making suicide the third most common cause of death worldwide and the second most common cause of death among those 15 to 29 years old. Furthermore, the suicide rate worldwide for men and women increases above the age of 70. In addition to this age–suicide correlation, gender plays an important role as another factor influencing suicide-related attitudes: the suicide rate among men is 1.8 times higher than among women. Women, however, have a higher number of suicide attempts, suicide-related thoughts, and suicide plans compared to men [2,3].

For the survivors of a suicide attempt as well as for the social environment (e.g., relatives or witnesses of suicides or suicide attempts), the effects are often psychologically stressful and associated with serious social consequences long after the suicide or suicide attempt took place. It should be emphasized that suicidal behavior is associated with the highest social stigmatization risk for those affected as well as for their relatives [4]. In addition, the financial cost of mental care and treatment of suicidality is high for the health system [3]. Accordingly, suicidality should not only be taken into account in terms of its epidemiological relevance, but also in particular because suicidality is highly stigmatized—comparable to the stigma of people with mental disorders [5]. According to the conceptualization of Link and Phelan [4], stigma is basically the result of a combination of five components: labeling, stereotyping, separation, status loss, and discrimination. In terms of the stigma of suicide, this would mean that characteristics of suicidal people would be perceived by the “non-suicidal” society as different. On that basis, stereotypical views towards suicidal people, such as “people with suicidal tendencies have weak wills”, would be formed. This would lead to negative emotional reactions towards this group (e.g.,: “I’m annoyed of suicidal people”) [4,6,7]. Since the treatment of suicidal patients is performed by medical and psychotherapeutic professionals, stigmatization of suicidality by the aforementioned professionals can have far-reaching effects: suicidal people often avoid seeking professional help out of shame and fear of resentment, which in turn increases the risk of suicidality [8,9]. Concerning the avoidance of seeking professional help, stigmatization of suicide by both the non-psychotherapeutic medical personnel (general practitioners, rescue workers, nursing staff or ward physicians, etc.) who often have the first direct (initial) contact with suicidal patients is highly relevant as well as the stigmatization of suicide by the psychotherapeutic medical personnel (e.g., psychiatrists and psychotherapists). This so-called structural stigmatization, which is defined as societal-level conditions, cultural norms, and institutional policies that constrain the opportunities, resources, and wellbeing of the stigmatized on the part of medical professionals can impede the success of the treatment of affected patients [5,10]. Subsequently, structural stigmatization can be followed by serious negative consequences for those affected in regard of their health and social lives.

It is also known that suicide-related stigmatization is subject to gender-specific differences, showing higher stigmatization of suicide by men compared to women. It has been speculated that stereotypical gender roles are the cause of this increased stigmatization, which could also cause men to avoid seeking professional help more than women [11]. This could also possibly lead to a greater understanding of the causes of suicidality on the part of women. For example, the study by Fox, Millner, Mukerji and Nock [12] in the United States found that more men than women die from suicide attempts. This finding is consistent with World Health Organization (WHO) (2019) data, which states that 1.8 times more men than women die by suicide each year worldwide. Suicide attempts without a lethal outcome, on the other hand, are committed mostly by young women [13]. Conversely, men report fewer normalizing tendencies towards suicide than women, such as understanding people who commit suicide or fantasizing and thinking about suicide, while women also make more suicide plans and attempts [14]. This phenomenon has become known among experts as “The Gender Paradox in Suicide” [15]. These results are congruent with the figures of the Federal Statistical Office (2020) for Germany, which also indicate that the largest share of completed suicides (76%) is committed by men. Therefore, the following question can be asked: to what extent has gender influenced the stigmatization of suicidal people? However, there were contradicting findings regarding age and the stigma of suicide. Nevertheless, it can be seen that the probability of an accomplished suicide increases with age. This is possibly an indication of decreasing stigmatization and greater normalization, or even glorification, of suicide (in the sense of admiring and exalting suicide) with people of an older age [16,17,18,19]. In summary, there are various factors influencing the stigmatization of suicide. Additionally, social exclusion of those affected and structural stigmatization counters adequate treatment. These factors can, in turn, favor suicidality in the sense of a vicious circle. Due to the serious consequences of the combination of stigma and suicidality, the aim of this study was to explore as to whether the stigma of suicide is less prevalent in already-practicing members of the health system as well as in future members of the health system, who are still in training, than in the general population. The medical staff, as well as the medical staff still in training, were compared to the general population on the premise that (future) medical staff show lower suicide stigmatization as well as a higher understanding of the causes of suicide due to professional training and the teaching of the relevant educational content. Trainees in the health system should at least distinguish themselves from the general population, but could possibly still have a higher stigmatization tendency than already working and thus more experienced members of the health system such as trained psychotherapists. Furthermore, the influence of the factors gender and age in terms of the stigmatization of suicide was examined. The influence of these factors should be lower among (future) health system members, because age and gender differences should be leveled out due to professional training. Since women make greater use of psychotherapy to cope with crises than men, there may be a gender difference in the understanding of the causes of suicidality [2,14]. Given the findings of the current literature review, women report more suicidal ideation and make more suicide plans as well as attempts than men; there may be a greater normalization or even glorification of suicide on the part of women. Furthermore, possible age and gender differences between the groups—related to stigmatization and understanding of causes as well as the normalization/glorification of suicidality—will be considered. The preceding assumptions led to the following questions: (1) does the general population show a higher level of stigmatization towards suicidality and also a lower understanding of the causes of suicidality than medical professionals and trainees? (2) Do men stigmatize suicide more than women and do women show more understanding of the causes of suicide as well as normalization or even glorification of suicidality, and do these gender differences show up least in the group of professionals, more in the group of medical trainees and most in the group recruited from the general population? (3) Is there an age effect towards lower levels of stigma and higher levels of normalization/glorification of suicidality with age, and is this more evident in the general population than among trained professionals?

## 2. Materials and Methods

An online survey was conducted, which explored the stigmatization of suicide (e.g., “people who commit suicide are pathetic”), understanding of causes leading to suicide (e.g., “people who commit suicide are depressed”) as well as “normalization/glorification” of suicide (e.g., items as “understandable” for normalization and “noble” for glorification). The survey lasted approximately 10 min and included questionnaires assessing suicide stigma (SOSS) and a range of demographics in order to assign the participants to the following three groups: general population (who reported having no professional contact with suicidal people), already practicing medical professionals (who have stated that they are in direct contact with suicidal people (general doctors, rescue workers, nursing staff or ward physicians, etc., as well as psychiatrists, psychologists and psychotherapists)) and future medical professionals (who are still in training to become the aforementioned professions). Informed consent was obtained from everyone in accordance with the Declaration of Helsinki. In addition, according to Oerter and Montada [20], three age groups were formed in order to be able to capture age effects: 18–35 years (young adulthood), 36–65 years (adults) and over 65 years (high adulthood). Due to the COVID- 19 pandemic, participants were recruited online through social media, online forums, and test-sharing platforms as well as via direct email contact to registered psychiatrists and psychotherapists. The online recruitment was targeted to individuals aged 18 and older living in Germany. The required sample size for a planned power of 1 − ß = 0.95 (two-tailed, α = 0.05, d = 0.06) was calculated with G*Power [21]. The a priori calculation resulted in a total sample size of 177 participants to be projected for a planned MANCOVA. A total of 779 participants fully completed the survey. The distribution of this overall sample into the three study groups as well as the gender distribution within the three study groups are shown in Table 1. All study participants ranged in age from 18 to 82 years (M = 32.15 years; SD = 13.012 years). Table 1 shows the respective numbers of participants for each of the three age groups as well as the age distribution within the groups. It was inevitable that the three groups would have different mean ages, as medical trainees are usually younger than medical professionals. Age was therefore controlled as a covariate in the statistics.

Table 1 shows the individual sample sizes of the three age groups as well as the two gender groups for each of the three study groups and the respective sizes of the respective total sample in the respective number and as a percentage of the total sample. *n* = number of participants in the respective group.

### 2.1. Used Psychological Testing Procedures

The SOSS [22] was used to assess the stigmatization of suicide. This 58-item scale was shown to have a three-factor structure, with the primary factor assessing stigma toward people who die by suicide, a factor of attributing suicide to isolation or depression, which reflects the understanding of causes and a “normalization/glorification” factor [22]. All three factors had strong internal consistency (α = 0.90). Each item consists of a one-word descriptor of a person who dies by suicide, rated on a 5-point Likert scale from (1) strongly disagree to (5) strongly agree. The subscales of the SOSS were summarized by calculating the mean response to all items on the subscale, ranging from 1 to 5 [22].

### 2.2. Analysis

In order to verify the comparability of the groups, a chi^2^ test was calculated; since the expected cell frequencies were smaller than five in each case, the exact test according to Fisher was interpreted for both the gender and the age groups [23]: here, a statistically significant correlation between gender and group membership (*p =* 0.045) as well as between age group and group membership (*p* < 0.001) was shown. Consequently, the genders and age groups in the main groups are statistically significantly unequally distributed, with the consequence being that both the “age” and “gender” of the subjects had to be controlled as covariates. To test for differences between the groups, multi-factorial covariance analysis (MANCOVA) was applied, controlling for age and gender. The significance level was set at *p* ≤ 0.05. Finally, partial eta^2^ indicates the effect size. Thereby, partial eta^2^ of 0.01 represents a small effect, 0.06 a medium effect and above 0.14 is a strong effect [24]. Post hoc pairwise comparisons were made using the Bonferroni calculation to determine between which pairs of groups is a statistically significant difference in the sum scores. For mathematical reasons, post hoc testing was preferred to the calculation of a priori contrasts, since the former is considered more effective, especially with large samples, as is the case here [25]. According to [26], the results of the multivariate tests were evaluated according to the “Pillai trace criterion”, as this procedure is considered conservative. All analyses were performed with the statistics software SPSS 25.

## 3. Results

The pairwise comparisons showed a statistically significant difference (Bonferroni-corrected *p* < 0.001) in the sum score values of the “stigmatization scale” between the group of the general population and the medical professionals, as well as between the group of medical trainees and medical professionals (Bonferroni-corrected *p* < 0.001) under control of the two covariates (age and gender) with a medium effect size η^2^ = 0.076. Regarding the pairwise comparisons of the “isolation/depression scale” of the SOSS, there was no statistically significant difference in the sum score values for any of the three groups with low effect size η^2^ < 0.001 (see Table 2).

Table 2 shows the statistical results of the post hoc comparisons for the sum scores of the two Bonferroni scales (“stigmatization” and “isolation/depression”) achieved by the three study groups, based on the estimated marginal means.

The comparison of the responses to the “stigmatization scale” between the general population, medical professionals and medical trainees revealed that the medical professionals had a statistically significant higher level of stigmatization of suicide than the members of the other two groups. The results indicate a higher level of stigmatization among health professionals compared to trainees and the general population. However, no differences in the responses to the isolation/depression scale were evident between the three groups, indicating an equally prevalent understanding of the causes of suicide.

Regarding the gender differences in the responses to the scales “stigmatization”, “isolation/depression” and “normalization/glorification” of the SOSS, the group of men showed a higher level of stigmatization with a low effect size η^2^ = 0.038, while the women had a higher mean score in terms of understanding of the causes of suicide (“isolation/depression” scale) at a low effect size η^2^ = 0.006 and a more pronounced normalization/glorification tendency in their responses with a weak effect size η^2^ = 0.011 (Table 3).

Table 3 shows the statistical results of the post hoc comparisons for the sum scores of the three Bonferroni stigmatization, isolation/depression and normalization/glorification achieved by the two gender groups, based on the estimated marginal means. 

The pairwise comparisons to investigate different gender responses between the groups of the general population, medical professionals and medical trainees showed a statistically significant difference in the sum scores of the “stigmatization scale” between the genders of the group of the general population (Bonferroni-corrected *p =* 0.025), with higher sum scores for men. There were also higher sum scores for stigmatization between the genders of the group of medical professionals (Bonferroni-corrected *p* < 0.001), with higher sum scores for women at a high effect size η^2^ = 0.110. For “isolation/depression scale”, a pairwise comparison after Bonferroni correction revealed a statistically significant difference within the group of the general population (Bonferroni-corrected *p =* 0.034), with higher sum scores for woman with a low effect size η^2^ = 0.013. Furthermore, there was a significant difference in the sum score values of the “normalization/glorification scale” between the genders of the medical professionals (Bonferroni-corrected *p =* 0.43) at a medium effect size η^2^ = 0.078, with higher sum score values for men (see Table 4).

Table 4 shows the statistical results of the post hoc comparisons for the sum scores of scales (“stigmatization”, “isolation/depression” and “normalization/glorification”) according to Bonferroni achieved by the gender groups in the respective subgroups, based on the estimated marginal means.

In summary, there were statistically significant gender differences in the levels of stigmatization among both professionals and the general population. However, the gender differences proved to be different, because although the level of stigmatization was higher among males in the general population, it was higher among female medical professionals. Regarding the “isolation/depression” scale, there was a statistically significant mean difference in the sum scores between the genders of the general population group (*p =* 0.034) with higher scores on the female side. Another significant difference in mean scores was found in the “normalization/glorification” scale, with lower scores on the part of the male medical professionals. The evaluation showed no differences in normalization/glorification tendencies among the male participants across all groups, suggesting a particularly low normalization/glorification score on the part of female medical professionals.

To capture possible age differences in response to the “stigmatization” and “normalization/glorification” scales, pairwise comparisons showed a statistically significant difference (*p* < 0.001) with a medium effect size η^2^ = 0.076 in the sum score values of the “stigmatization” scale between the age groups “18–35 years” and “36–65 years”, with higher sum scores for the “36–65 years” participants. For the “normalization/glorification” scale, no statistically significant pair comparison was evident after Bonferroni correction with a weak effect size η^2^ = 0.004. Thus, no statistically significant rise in the scores for stigmatization and normalization/glorification with increasing age was evident (Table 5). Since the age category over “65 years”, with a total of only 14 participants, was clearly underrepresented compared to the other two age groups, this age category was not included in the calculation. (Table 5).

Table 5 shows the statistical results of the post hoc comparisons for the sum scores according to Bonferroni for the scales “stigmatization” and “normalization/glorification” obtained by the two age groups, based on the estimated marginal means. 

In respect to different age-related responses in the comparison between the general population and health professionals, pairwise comparisons according to Bonferroni showed statistically significant differences: regarding the medical professionals, the pairwise comparisons revealed a statistically significant difference (*p* < 0.001) with a high effect size η^2^ = 0.160 in the sum score values of the “stigmatization” scale between the age groups “18–35 years” and “36–65 years”, with higher sum scores for the 36–65-year-old participants. On the contrary, the comparisons of the age groups “36–65 years” and “over 65 years” as well as the age groups “18–35 years” and “over 65 years” showed no significant difference in the sum score means. Within the general population, there were no significant effects between all age groups regarding the “stigmatization” scale. For the “normalization/glorification” scale, no significant main effect was evident between all age groups in the pairwise comparison, either within the general population or among the medical professionals (η^2^ = 0.077) (Table 6).

Table 6 shows the statistical results of the post hoc comparisons for the sum scores of scales “stigmatization” and “normalization/glorification” according to Bonferroni achieved by the two age groups within the two study groups, the general population and medical professionals, based on the estimated marginal means. 

## 4. Discussion

The present study investigated the differences in stigmatization of suicidality between the general population, health professionals and future health professionals still in training, also with regard to possible gender and age effects.

Our first question was: does the general population show a higher level of stigmatization towards and also a lower understanding of the causes of suicidality than professionals trained in dealing with suicidality and trainees/students?

The results are contrary to our hypothesis. It should be emphasized that the medical professionals counterintuitively stigmatized significantly more than the other two groups, which showed no differences in the level of stigmatization. The latter result is also surprising, because the “untrained” general population did not show any differences in comparison with the students/trainees of the health system, whose training should at least have introduced them to this specific topic to a greater extent. The three groups did not differ significantly in their understanding of causes, which suggests an evenly prevailing understanding of the reasons for suicidality in the groups studied. Our results provide a hint that the general population group already had as high an understanding of causes, and thus some kind of “specialist knowledge” about suicidality, as the professionals, and that both groups were at a common high level (>75% of the maximum achievable score). The higher stigmatization tendency of the professionals could indicate that—in the course of practical professional experience with suicidality—factors come into play that reinforce the stigmatization tendencies.

Our second question was: do men stigmatize more than women and do women show more understanding as well as normalization/glorification of suicidality, and do these gender differences show up least in the group of professionals, more in the group of trainees and students of the health system and most in the group from the general population?

This task of the study can be answered as follows: the results show the assumed gender difference in stigmatization, understanding of causes, and normalization/glorification across all groups, which means that this first part of the question can be answered in the affirmative. In view of the gender relations within the study groups, a much more differentiated picture emerges: with regard to the level of stigmatization, female medical professionals showed a higher level than men and there were no gender differences in the trainee group in this regard. Within the general population, on the other hand, a higher level of stigmatization could be seen among men. Regarding the understanding of causes, the assumed gender difference effect was shown exclusively in the general population group, with lower understanding of causes on the part of men. The same applied to the level of normalization/glorification, which only showed gender differences among professional staff. In the latter, the female participants showed lower normalization/glorification tendencies.

Question 3 was: is there an age effect towards lower levels of stigma and higher levels of normalization/glorification of suicidality with age, and is this more evident in the general population than among trained professionals? All components of the question can be answered contrary to our hypothesis, as there was neither less stigmatization nor more normalization/glorification with higher age. It should be noted, however, that within the general population the sample of “over 65-year-old” participants was clearly underrepresented with *n* = 3 participants compared to the other two age groups (“18–35-year-old” with *n* = 231 and “36–65-year-old” with *n* = 94). The same applies to the group of the medical professionals, in which the “over 65” participants were in the minority, with *n* = 11 participants (compared to “18–35-year-old” with *n* = 64 and the “36–65-year-old” participants with *n* = 99). Therefore, the age group was not taken into account in the calculation. However, apart from the MANCOVA, which ensured the control of gender as a confounding variable, a correlative calculation was also carried out as an additional test of age and stigmatization tendency as well as normalization/glorification. (r = 0.259, *p* < 0.001; r = 0.011, *p =* 0.380). The reason for the higher level of male stigmatization could be speculated to be the prevailing social role model: while stereotypical male gender roles are characterized by a higher attribution of qualities such as “strength”, “independence”, “risk-taking behavior”, etc., stereotypical female gender roles are characterized on the one hand by positive attributions such as “empathy” and “emotionality”—but also by negative attributions such as “weakness” and “incompetence” [27,28,29]. Furthermore, negative “typically female” attributions are also socially attributed to men who appear “feminine”, as well as suicidal persons or persons suffering from suicidal persons or persons suffering from mental disorders [30]. Suicidal men are socially stigmatized more than suicidal women [11]. Here, a stronger stigmatization of suicidality by men serves as a social demarcation from men who are considered to be “weak” [31,32,33,34]. As a result, however, the socially strong manifestations of these gender stereotypes have the effect of preventing men from seeking professional help in the case of suicidality and depression. Women, on the other hand, make more frequent and quicker use of professional help to cope with crises. An important factor here is the lower social stigmatization of depressiveness or suicidality among women [2,11]. The more pronounced understanding of the causes of suicidality among women could also play a role here: if knowledge about the causes of suicidality is available, stigmatization also decreases [8], and thus probably also an inhibition threshold to seek help. Furthermore, the tendency to normalize/glorify suicide, which is more pronounced among the women of the total sample, could also contribute to the explanation of their lower level of stigmatization shown in the total sample. This would correspond to the romanticized approach to suicidality that is prevalent in Japan, for example, which goes hand in hand with little stigmatization but high suicide rates [35,36]. The assumption of an involvement of normalization and glorification trends in the reduced stigmatization tendency by women seems to be congruent with the numbers of the Federal Statistical Office regarding the majority of suicide attempts committed by women [37].

In view of the present results, the question arises as to why women among the trained professionals show a higher level of stigmatization than men and why this effect is reversed within the general population. As already shown, more women than men attempt suicide and are also more likely to seek therapeutic treatment. In the stationary clinical setting, for example, women who show self-harming behavior and/or suicidal tendencies in the context of a borderline personality disorder are overrepresented compared to men [38]. Furthermore, there are also more women than men working in caring or “helping professions”, which include the group of professional staff considered here [39]. Thus, in view of the current state of studies, it can be assumed that more female suicidal patients present themselves to the female professionals considered here—could the stigmatization of suicidal people thus be linked to hostile attitudes towards their own gender? Further dedicated research would be necessary to clarify this question, but there are already findings on hostility among women towards female victims of sexual abuse: they are blamed by women for the crime, as well as it being insinuated that they were “only trying to attract attention” with their victim status [40]. This creates a demarcation between the women’s own group and the “foreign group” of rape victims, which serves to maintain a positive self-image [41]. These demarcation mechanisms could possibly also have led to a greater stigmatization of suicidality by women in the health system. Since SOSS does not specify in its instructions which kind of suicidal person was chosen by the participants as the basis for their answers, it can only be speculated here whether a prototypical female (borderline) image of a suicidal person was generated, which in turn could have triggered the described demarcation mechanisms among female medical professionals. Furthermore, the intergroup comparison showed that all three groups of men did not differ from each other in their tendency to stigmatize, but the women in the medical professionals group showed higher stigmatization than all other gender groups in each group. Women in the general population and students/trainees, on the other hand, showed lower levels of stigmatization than men in the general population as well as within the medical professionals. These findings could be interpreted as an indication that women only begin to stigmatize more strongly through professional contact, while men’s level of stigmatization remains constant. It can thus be assumed that the aforementioned demarcation mechanisms of the female medical professionals towards the suicidal female patients who were perceived as exhausting and manipulative led to the higher level of stigmatization [38]. The stigmatization due to possible demarcation mechanisms could be greater among the female medical professionals compared to male medical professionals since males could be already used in having demarcation mechanisms towards the group of the “weak”. One could assume that the reason for this is that men are routinized to maintain their socially anchored and demanded positive self-image as a “strong man”. Women, on the other hand, could possibly be confronted for the first time in a professional context with the need to distinguish themselves from the patients concerned. This could then lead to reactive overcompensation, which leads the women in the professional staff group to demonstrate higher stigmatization. In accordance with their higher stigmatization tendency, the female medical professionals also show lower normalization/glorification tendencies compared to the men of the professional group. Thus, only in this group of female participants was there a negative, highly pronounced correlation between the “stigmatization” and “normalization/glorification” scales (r = −0.810, *p* < 0.001). Within the general female population (r = −0.256, *p* < 0.001) and the group of female students and trainees (r = −0.006, *p =* 0.465), on the other hand, there were only weak correlations with regard to the responses to the two scales. As a result, the more pronounced the stigmatization, the lower the normalization/glorification in the group of female professionals. This finding suggests a connection between the two values, which may then be influenced by a third variable of experience with suicidal patients. Possible gender-specific demarcations and/or aversive experience values due to the activity could also play a role here. The results for the examination of age differences show a tendency within the professional group towards higher stigmatization with increasing age. In this context, Norheim et al. [8] found that suicide-related attitudes could be dependent on the professional training of medical staff. This was associated with “more positive attitudes”, which led to a less severe structural stigmatization of suicidal people by treating professionals. This was shown, for example, in a decrease in agreement with the statements that suicide “cannot be justified” or that suicide “should not be talked about”. Interestingly, there was a statistically significant age effect among professional staff members in the age groups “18–35 years” and “36–65 years”, with higher stigmatization tendencies among the older participants. Corresponding to actual facts, this could also reflect a “training/practice” effect.

### Limitations

In general, it can first be stated that the sample obtained showed a majority of women in the group of the general population, which can be attributed to the type of recruitment: due to the COVID-19 pandemic situation with contact blocks, only online recruitment was possible. Many of the participants therefore took part via internet forums whose target group is primarily women, while recruitment from internet forums that tend to be male-dominated (e.g., concerning certain hobbies) was not very successful within the given time schedule for our study. Furthermore, due to only low recruitment rates from internet forums, healthcare professionals had to be additionally recruited by email, which nevertheless yielded a relatively low number of participants from the healthcare sector. In addition, the proportion of students/trainees is largely represented by Medical School of Hamburg students. This means that a certain place of education is overrepresented among the students, so that the level of education may have a low variance. Furthermore, only very few subjects in the age group over 65 years could be recruited, so that they could partly not be included in analyses. As mentioned, recruitment due to the COVID-19 pandemic took place at a time of increased exit and contact restrictions. This triggered psychological distress in the population, which could have led to an increased use of therapeutic help and thus to a strain on the members of the professional staff [42]. These circumstances could have influenced the response reactions in the direction of stronger stigmatization. Another limiting factor could be found in the nature of the research method: the SOSS is designed to process data based on subjective beliefs about suicidal people. However, it remains unknown which prototypical characteristics are present in the participants’ conceptions. Therefore, it is possible that, for example, the professionals reacted to the questionnaire on the basis of the idea of a prototypical suicidal borderline patient, since these patients in particular are often treated in the inpatient setting under the impression of frequent and sometimes manipulative suicidality, and seem particularly impressive and people-/labor-intensive when they appear in the clinical setting, even with only a few contacts [38]. This may have led to a bias through sexist location and thus caused the professional per se, and/or the female members of the professional staff, to tend to react in a stigmatizing way. In addition to these limitations in view of the significance of the study, distortions due to the data obtained cannot be completely ruled out: for example, the calculations show low effect sizes in some cases, which may mean that existing differences between the compared groups could not be revealed. This concerns:

- possible, undetected differences in the understanding of causes between the three study groups and between the gender groups of the two groups of professional staff and trainees/students. If this difference were manifest, one could assume a lower understanding of causes in the respective less educated groups. However, this would not affect the interpretations presented and the main statements would remain.
- a possible, undetected difference between the gender regarding normalization/glorification tendencies in the overall sample. A higher normalization/glorification effect could be assumed in view of the further results within the total sample, However, concerning the interpretation produced, the gender differences evident in the professional staff group with a high effect size appear to be much more important.
- a possible, undetected effect in differences in the tendency towards stigmatization and normalization/glorification between the two older age groups. Here, an effect could be assumed that replicated the evident differences between the younger and middle age groups (at least recognizable in the professional staff group). This would not have to lead to any significant changes in the interpretations.

## 5. Conclusions

The results of the present work indicate that professionals have an increased tendency to stigmatize suicidal patients. This would make it necessary to question the current training situation of the prospective medical professionals and the handling of suicidality by already practicing professionals. Therefore, the findings of the study could have implications for the training system, but also for the further training of professionals already working in the field and required adjustments to the teaching. In this context, the risks of inadequate treatment and the associated structural stigmatization should be explicitly addressed, since (at least in view of the results on the scale “isolation/depression”) there is already factual knowledge about the causes of suicidality. It is also possible that the gap between theoretical content and actual practical experience with suicidality is too large and is not sufficiently compensated for in the context of supervision, intervision and further training. Here, the dedicated training of established or future treatment providers of suicidal people could also be useful in order to enable an appropriate treatment and to prevent suicide due to structural stigmatization.

## Figures and Tables

**Table 1:** Sample sizes of the three age groups within the study groups, sample sizes of the two gender groups for the respective study groups.

Gender	*n* (Percentage of Overall Sample) Gender Groups		*n* (Percentage of Overall Sample) Age Groups
General Population			
Male	90 (11.6)	18–35 years	231 (29.5)
Female	238 (30.6)	36–65 years	94 (12.1)
		over 65 years	3 (0.4)
Total	328 (42.2)	Total	328
Medical professionals			
Male	108 (13.8)	18–35 years	64 (8.2)
Female	66 (8.5)	36–65 years	99 (12.7)
		over 65 years	11 (1.4)
Total	174 (22.3)	Total	174
Medical professionals in training			
Male	56 (7.2)	18–35 years	272 (34.9)
Female	221 (28.3)	36–65 years	5 (0.6)
		over 65 years	0 (0)
Total	277 (35.5)	Total	277
Overall sample			
Male	254 (32.6)	18–35 years	567 (72.8)
Female	525 (67.4)	36–65 years	198 (25.4)
		over 65 years	14 (1.7)
Total	779 (100.0)	Total	779 (100.0)

**Table 2:** Results of the pairwise comparisons according to Bonferroni of the three study groups post hoc of the MANCOVA regarding the sum scores in the “stigmatization scale” and “isolation/depression scale” of the SOSS.

(I) Group	(J) Group	Mean Difference (I-J)	Standard Error	Significance Level	95% Confidence Interval for the Difference	
					Lower Limit	Upper Limit
	Stigmatization Scale					
General Population	Medical professionals	−0.693 **	0.091	0.000	−0.912	−0.474
	Medical trainees	0.061	0.080	1.000	−0.130	0.252
Medical professionals	General Population	0.693 **	0.091	0.000	0.474	0.912
	Medical Trainees	0.755 **	0.107	0.000	0.497	1.012
Medical Trainees	General Population	−0.061	0.080	1.000	−0.252	0.130
	Medical Professionals	−0.755 **	0.107	0.000	−1.012	−0.497
	Isolation/Depression Scale					
General Population	Medical professionals	−0.028	0.099	1.000	−0.266	0.210
	Medical trainees	−0.042	0.087	1.000	−0.249	0.166
Medical Professionals	General Population	0.028	0.099	1.000	−0.210	0.266
	Medical trainees	−0.014	0.116	1.000	−0.293	0.266
Medical trainees	General Population	0.042	0.087	1.000	−0.166	0.249
	Medical professionals	0.014	0.116	1.000	−0.266	0.293

** *p* < 0.01.

**Table 3:** Results of the pairwise comparisons according to the Bonferroni test of the two gender groups (post hoc of the MANCOVA) regarding the sum scores for the “stigmatization”, “isolation/depression” and “normalization/glorification” scales of the SOSS.

(I) Group	(J) Group	Mean Difference (I-J)	Standard Error	Significance Level	95% Confidence Interval for the Difference	
					Lower Limit	Upper Limit
stigmatization						
male	female	0.434 **	0.078	0.000	0.281	0.588
female	male	−0.434 **	0.078	0.000	−0.588	−0.281
isolation/depression						
male	female	−0.182 *	0.082	0.026	−0.343	−0.022
female	male	0.182 *	0.082	0.026	0.022	0.343
normalization/glorification						
male	female	−0.244 **	0.082	0.003	−0.406	−0.083
female	male	0.244 **	0.082	0.003	0.083	0.406

* *p* < 0.05. ** *p* < 0.01.

**Table 4:** Results of the pairwise comparisons according to the Bonferroni test of the two gender groups of the three study groups (post hoc of the MANCOVA) with regard to the sum scores for the “stigmatization”, “isolation/depression” and “normalization/glorification” scales of the SOSS.

(I) Group	(J) Group	Mean Difference (I-J)	Standard Error	Significance Level	95% Confidence Interval for the Difference	
					Lower Limit	Upper Limit
	Stigmatization scale					
General population male	General population female	0.356 *	0.113	0.025	0.024	0.688
Medical professionals male	Medical professionals female	−0.621 **	0.143	0.000	−1.041	−0.200
Medical trainees male	Medical trainees female	0.195	0.136	1.000	−0.207	0.596
	Isolation/Depression scale					
General population male	General population female	−0.374 *	0.122	0.034	−0.733	−0.014
Medical professionals male	Medical professionals female	0.062	0.155	1.000	−0.393	0.517
Medical trainees male	Medical trainees female	0.067	0.148	1.000	−0.367	0.502
	Normalization/Glorification scale					
General population male	General population female	−0.191	0.119	1.000	−0.542	0.160
Medical professionals male	Medical professionals female	0.451 *	0.151	0.043	0.007	0.896
Medical trainees male	Medical trainees female	0.011	0.144	1.000	−0.414	0.435

* *p* < 0.05. ** *p* < 0.01.

**Table 5:** Results of the pairwise comparisons according to the Bonferroni test of the two age groups (post hoc of the MANCOVA) regarding the sum scores for the scales “stigmatization” and “normalization/glorification” of the SOSS.

(I) Group	(J) Group	Mean Difference (I-J)	Standard Error	Significance Level	95% Confidence Interval for the Difference	
					Lower Limit	Upper Limit
	“Stigmatization” Scale					
18–35	36–65	−0.617 **	0.078	0.000	−0.803	−0.431
36–65	18–35	0.617 **	0.078	0.000	0.431	0.803
	“Normalization/Glorification” Scale					
18–35	36–65	0.070	0.082	1.000	−0.128	0.267
36–65	18–35	−0.070	0.082	1.000	−0.267	0.128

** *p* < 0.01.

**Table 6:** Results of the pairwise comparisons according to the Bonferroni test of the two age groups within the general population and the medical professionals (post hoc of the MANCOVA) regarding the sum scores for the “stigmatization” and “normalization/glorification” scales of the SOSS.

(I) Age Group per Group	(J) Age Group per Group	Mean Difference (I-J)	Standard Error	*Sig.*	95% Confidence Interval for the Difference	
					Lower Limit	Upper Limit
		Skala 1				
Generals: 18–35	Generals: 36–65	−0.098	0.110	1.000	−0.442	0.246
Generals: 36–65	Generals: 18–35	0.098	0.110	1.000	−0.246	0.442
Medicals: 18–35	Medicals.: 36–65	−0.727 **	0.144	0.000	−1.178	−0.276
Medicals: 36–65	Medicals: 18–35	0.727 **	0.144	0.000	0.276	1.178
		Skala 3				
Generals: 18–35	Generals: 36–65	−0.270	0.117	0.608	−0.638	0.098
Generals: 36–65	Generals: 18–35	0.270	0.117	0.608	−0.098	0.638
Medicals: 18–35	Medicals: 36–65	0.237	0.154	1.000	−0.246	0.721
Medicals: 36–65	Medicals: 18–35	−0.237	0.154	1.000	−0.721	0.246

** *p* < 0.01. (General population = “Generals”, Medical professionals = “Medicals”).

## Data Availability

The data presented in this study are available on request from the corresponding author.
